# Deceased Convicts

**Published:** 1856-05

**Authors:** 


*Deceased Convicts.*—A bill was recently introduced into the Legislature of New York, to amend the laws relative to the disposition of the bodies of deceased convicts, by giving to the Medical Faculty of the University of New York, one-third of the "subjects" furnished by the State from her prisons. —*Ibid.*

